# Clinical application of immune repertoire sequencing in solid organ transplant

**DOI:** 10.3389/fimmu.2023.1100479

**Published:** 2023-02-14

**Authors:** Paaksum Wong, Davide P. Cina, Karen R. Sherwood, Franz Fenninger, Ruth Sapir-Pichhadze, Constantin Polychronakos, James Lan, Paul A. Keown

**Affiliations:** ^1^ Department of Medicine, University of British Columbia, Vancouver, BC, Canada; ^2^ Department of Urologic Sciences, University of British Columbia, Vancouver, BC, Canada; ^3^ Department of Pathology and Laboratory Medicine, University of British Columbia, Vancouver, BC, Canada; ^4^ Department of Medicine, Division of Nephrology, McGill University, Montreal, QC, Canada; ^5^ Department of Epidemiology, Biostatistics and Occupational Health, McGill University, Montreal, QC, Canada; ^6^ Department of Pediatrics, The Research Institute of the McGill University Health Centre and the Montreal Children’s Hospital, Montreal, QC, Canada

**Keywords:** solid organ transplant, alloimmunity, lymphocyte receptor sequencing, B cell receptor (BCR), T cell receptor (TCR)

## Abstract

**Background:**

Measurement of T cell receptor (TCR) or B cell receptor (BCR) gene utilization may be valuable in monitoring the dynamic changes in donor-reactive clonal populations following transplantation and enabling adjustment in therapy to avoid the consequences of excess immune suppression or to prevent rejection with contingent graft damage and to indicate the development of tolerance.

**Objective:**

We performed a review of current literature to examine research in immune repertoire sequencing in organ transplantation and to assess the feasibility of this technology for clinical application in immune monitoring.

**Methods:**

We searched MEDLINE and PubMed Central for English-language studies published between 2010 and 2021 that examined T cell/B cell repertoire dynamics upon immune activation. Manual filtering of the search results was performed based on relevancy and predefined inclusion criteria. Data were extracted based on study and methodology characteristics.

**Results:**

Our initial search yielded 1933 articles of which 37 met the inclusion criteria; 16 of these were kidney transplant studies (43%) and 21 were other or general transplantation studies (57%). The predominant method for repertoire characterization was sequencing the CDR3 region of the TCR β chain. Repertoires of transplant recipients were found to have decreased diversity in both rejectors and non-rejectors when compared to healthy controls. Rejectors and those with opportunistic infections were more likely to have clonal expansion in T or B cell populations. Mixed lymphocyte culture followed by TCR sequencing was used in 6 studies to define an alloreactive repertoire and in specialized transplant settings to track tolerance.

**Conclusion:**

Methodological approaches to immune repertoire sequencing are becoming established and offer considerable potential as a novel clinical tool for pre- and post-transplant immune monitoring.

## 1 Principal mechanisms of alloantigen recognition

Transplant biopsy is the current gold standard for diagnosing rejection in solid organ transplant, combining histology with more detailed opportunities for cellular, molecular, and spatial biology evaluation of graft injury. But by virtue of its invasive nature, biopsy is normally employed only when clinical data raise diagnostic suspicion. Donor-derived cell-free DNA (dd-cfDNA) has shown potential as a biomarker for early detection of immune activation and serial surveillance (1). Novel solid-phase or cellular assays detecting anti-human leukocyte antigen (HLA) antibodies help to discriminate between cellular (CMR) and antibody mediated rejection (AMR). Unfortunately, current diagnostic methods are limited in utility and application, and typically only confirm rejection once significant organ damage has already occurred. We therefore require novel, less invasive and reliable assays for monitoring the alloimmune response in solid organ transplant to recognize immune events at the earliest possible time prior to the advent of irreversible organ injury. Such assays permit a more precise understanding of the biological mechanisms leading to graft injury, and the opportunity for earlier clinical intervention, mitigating rejection and improving graft outcomes.

Genomic analysis of the T and B cell receptor (TCR, BCR) repertoires promises to further our understanding of immune rejection and allows us to develop clinical biomarkers of graft injury for predicting short- and long-term graft outcomes (2, 3) T and B cells are an attractive target for study because they are cornerstones of the adaptive immune response in the post-transplant period and play key roles in alloreactivity and tolerance. T cells are responsible for cell-mediated rejection (CMR) as well as initiating donor-specific antibody (DSA) formation. In the latter, activated T helper cells trigger the differentiation of B cells and their production of DSAs, leading to antibody-mediated rejection (AMR) (4).

Two main processes underlie T cell-mediated allorecognition, both of which occur through the TCR. In the early post-transplant period, alloreactivity is thought to be driven primarily by direct recognition in which recipient T cells bind HLA-peptide complexes expressed on allogeneic cells. This response gradually transitions to the main driver of long-term allograft rejection (5, 6) considered to be indirect allorecognition, which occurs when recipient antigen-presenting cells (APCs) take up, digest and present donor HLA peptides in the context of self HLA to engage recipient T cells. Recipient T cells recognize this self HLA-foreign peptide complex through the TCR (5, 6) recruiting cytotoxic CD8 T cell responses and driving long-term alloimmune recognition (7). B cell-mediated allorecognition, an extension of this process, occurs *via* B cell receptor (BCR)-mediated recognition of an HLA-peptide complex presented by helper T cells. Once activated, the B cells mature and differentiate into antibody-secreting plasma cells or memory B cells. A single activated B cell may generate 5000 plasma cells in a week and up to 10^12^ antibodies per day (8). These antibodies, directed against structural epitopes on the allogeneic HLA, drive the more destructive AMR with often devastating consequences for the graft. A third less well described pathway is also described, termed the semi-direct pathway (9). In this pathway, recipient APCs uptake intact donor HLA *via* cell-cell contact (10) or extra cellular vesicles (11, 12) and present them on their cell surface to stimulate an alloreactive immune response. Alloantigen-specific recognition events trigger downstream activation of other adaptive immune cells that mount a coordinated antigen-specific response and generate immunologic memory.

Tolerance, a state of immune unresponsiveness to tissues and cells, consists of central and peripheral tolerance. Central tolerance is the deletion of self-reactive clones during negative selection in the thymus, while peripheral tolerance includes peripheral deletion, anergy, and Treg suppression. Operational tolerance can be defined as stable graft function without immunosuppressive treatments and the absence of graft rejection (13). However, it is rare in practice to be safely and completely withdrawn from immunosuppression, and reasons to do so may include non-compliance, malignancies, infections, or serious side effects. Tolerance in liver transplant is relatively frequent in SOTs, and biomarkers of tolerance have been previously described with tolerant patients having increased frequencies of peripheral CD4+CD25+FoxP3+ cells (14). In kidney transplant, recipients who received kidneys from young living donors have seen better tolerance outcomes (15). However, graft function may decrease without prior indication, highlighting the importance of and demand for routine non-invasive monitoring.

## 2 The T cell immune synapse and selective modulation of molecular signals

T cell allorecognition is among the earliest steps in the adaptive immune response to transplanted tissues. This process is tightly regulated and requires multiple signals acting in concert.

The first and most critical signal is T cell receptor (TCR) recognition of a cognate antigenic peptide presented on a class I or class II major histocompatibility complex (MHC). This interaction is stimulatory, and its strength depends on the density and affinity of the TCR-MHC interaction (16). Antigen analog-MHCs have been explored as TCR antagonists to inhibit antigenic peptide engagement to the TCR as a target for treating allergies or autoimmune diseases (17). However, few studies have investigated its application in transplant immunosuppression likely due to the unique specificities of individual TCRs which are different for every given recipient-donor pair. When compounded with the complexity of identifying potentially alloreactive MHC-TCR combinations, targeting TCR directly as a means of immunosuppression may be difficult.

**Figure 1:**
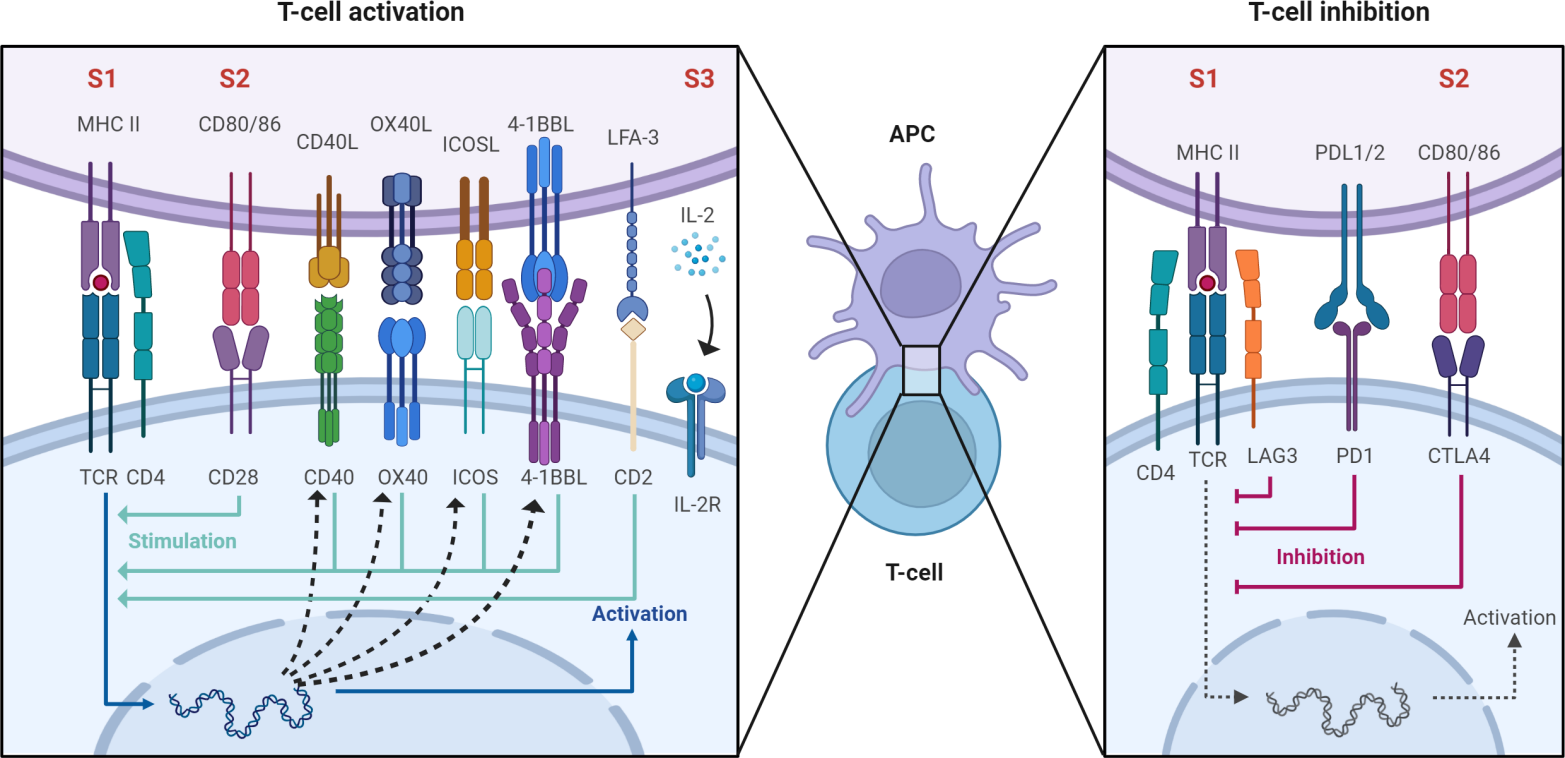
After antigen specific allorecognition *via* the antigen-specific TCR-MHC interaction (signal 1, labeled S1) second order signals are required for full T cell activation (S2 and S3). These involve engagement of immune checkpoints which are receptor-ligand pairs belonging to either the TNF or immunoglobulin superfamily. These act to either co-stimulate or co-inhibit the T cell response. Created with BioRender.com.

Once antigen specific recognition occurs, the second signal involves engagement of immune checkpoints which are receptor-ligand pairs belonging to either the TNF or immunoglobulin superfamily. These act to either co-stimulate or co-inhibit the T cell response upon TCR engagement thereby directing and tightly regulating the subsequent T cell response (**Figure 1**). Because of these properties, immune checkpoints are the target of a new generation of immunosuppressive medications. The most well-described checkpoint is CD28 which engages CD80/CD86 (B7) to stimulate T cell activation (18). CTLA4 is a co-inhibitory molecule that competes with CD28 to bind CD80/CD86 and inhibit T cell activation (18). Belatacept is a second generation CTLA4-Fc fusion protein, used in CNI-sparing immunosuppressive regimens, that binds to CD80/CD86 on APCs and blocks engagement of CD28 on T cells thereby inhibiting their activation (19). CD40L is another co-stimulatory checkpoint expressed on T cells that binds to CD40 (CD154) on antigen presenting cells and endothelial cells to stimulate T cell activation (20, 21). Inhibition of CD40 using a humanized monoclonal antibody was sufficient to prevent rejection in nonhuman primate renal transplants (22) but concerns of thromboembolic complications have slowed its translation to clinical use (23). OX40, ICOS, and 4-1BB are so-called ‘secondary checkpoints’ expressed on activated T cells that are involved in further stimulating T cell activation after initial engagement of CD28. OX40 binds OX40L and is potentially involved in co-stimulation blockade resistant allograft rejection as co-administration of belatacept and anti-OX40L prolongs renal allograft survival in non-human primates relative to either therapy alone (24). ICOS is upregulated upon T cell activation and binds to ICOSL on antigen presenting cells. This interaction facilitates T cell activation, differentiation, and effector functions although its blockade alone or in combination with belatacept is insufficient to prolong renal allograft survival in NHPs (25). 4-1BB is also upregulated upon T cell activation and binds 4-1BBL to preferentially stimulate the CD8 cytotoxic T cell response (26). More recently, interest has turned to CD2 which is a co-stimulatory molecule expressed on T cells and NK cells (27). CD2 binds LFA-3 to regulate positive selection during T cell development and stimulate antigen specific activation in naïve and memory T cells (28–30). While data on the role of CD2 in NK cell function is more limited, it plays a role in antibody-independent and -dependent cytotoxicity (31). Two agents targeting this axis have been tested in the setting of human clinical transplant: Alefacept which is an LFA-3 Fc fusion protein and siplizumab which is a humanized monoclonal antibody targeting CD2. Alefacept has been tested as an induction agent in standard renal transplant agent with equivocal success (32). Siplizumab has been tested for safety in standard human renal transplant (33) and used as part of a pre-conditioning regimen in humans receiving a combined kidney bone marrow transplant to induce tolerance (34, 35). LAG-3 recognizes peptide-bound MHC class II and inhibits T cell activation (36). Last, PD1 is an inhibitory checkpoint expressed on T cells that binds to PDL1 on target cells to block T cell activation. While modulation of this checkpoint has little utility in transplantation, it has been leveraged extensively in cancer immunotherapy (37).

TCR engagement and co-stimulation together lead to downstream activation of the calcineurin/NFAT, MAP kinase, and NF-kB pathways which in turn lead to the transcription of cytokines which provide the third signal for T cell proliferation and differentiation (38). Because all these inputs converge on the calcineurin/NFAT pathway, it is the target of the calcineurin-inhibitors (CNIs) Cyclosporin (39) and Tacrolimus (40) which are the current cornerstones of transplant immunosuppression.

Last, the third signal which occurs upon antigen specific TCR engagement and co-stimulation, is the evolution of cytokines to direct T cell proliferation and differentiation. The prototypical signal responsible for T cell proliferation is IL2 which binds the IL2 receptor (IL2R) on T cells. Downstream activation of the mechanistic target of rapamycin (MTOR) through IL2R favours the proliferation and differentiation of conventional T cells whereas IL2R engagement in the presence of MTOR inhibition favours the proliferation and differentiation of T regulatory cells through STAT5 signaling (41). The IL2R is targeted by basiliximab which is a commonly used form of induction therapy in transplant (42). While many other cytokines are involved in directing further T cell differentiation, an extensive discussion of this topic is beyond the scope of this review (43).

## 3 The B cell immune synapse and signaling

B cell signaling and activation is a fundamental step in the humoral immune response and in AMR. First, immature B cells released from the bone marrow circulate in the secondary lymphoid organs (spleen and lymph nodes) until an antigen is encountered (44). Recognition of antigen by the BCR triggers the formation of the immunological synapse where initial cell spreading occurs. Antigens gather into microclusters during this stage and signaling is enhanced at these sites (45). Next, a contraction phase begins where the antigen is concentrated in the centre of the synapse forming the central supramolecular activation cluster (cSMAC), which is surrounded by actin and adhesion molecule-rich peripheral SMAC (pSMAC) (46). B cells are unique in that they can both interact with and act as APCs; a function of BCR-antigen binding is triggering endocytosis and intracellular processing (47). These B cells present processed peptides on MHCII molecules to CD4+ T helper cells through the TCR which signals the T cell to express CD40L, IL-4, and IL-21, promoting B cell proliferation, class switching, and somatic hypermutation (48). Activated B cells may immediately differentiate into plasmablasts for early production of low affinity antibodies for a quick immune response. But they can also form a germinal centre where further somatic hypermutation and class switching occur, eventually differentiating into memory B cells or plasma cells which are both capable of producing higher affinity antibodies (49). Memory B cells are formed when follicular dendritic cells present antigens for extended periods of time, maintaining the germinal centre response.

B cells have also been targeted for immunosuppression in transplant. Rituximab is directed against CD20 on the B cell surface and has been used extensively in treatment of AMR as well as desensitization therapies (50). B cell activation and survival may also be modulated by epratuzumab, an anti-CD22 antibody that depletes naïve and transitional B cells while inhibiting B cell activation and proliferation (51).

## 4 Receptor rearrangement and sequencing

**Figure 2:**
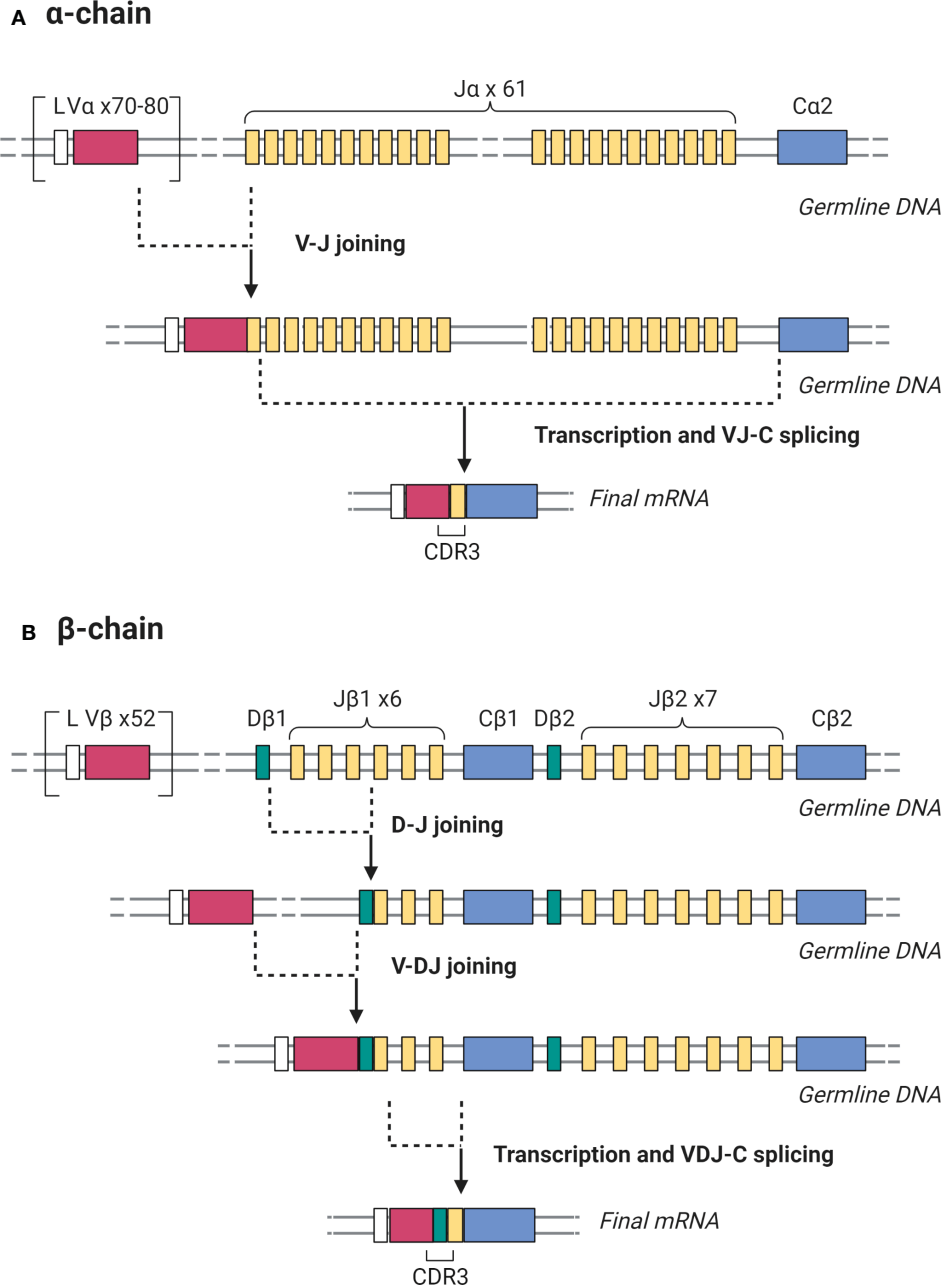
Somatic recombination of the germline DNA encoding TCR α and β chains during T cell development generates antigen-binding diversity. **(A)** For the TCR α chain, V-J rearrangement is followed by transcription and splicing to create a complete VJ-C mRNA. **(B)** For the β chain, D-J rearrangement is followed by V-DJ rearrangement, then transcription and splicing to create a complete VDJ-C mRNA. In both cases, the mRNA product is then translated to yield the protein receptor chains. The CDR3 region is the most variable portion of the TCR due to its location in the junction where V-J and V-D-J joining occurs in the α and β chains respectively. Created with BioRender.com.

As mentioned previously, antigen recognition by the TCR is the first crucial step in T cell activation and TCR diversity allows the adaptive immune system to respond to a multitude of antigenic stimuli. The TCR heterodimer consists of an α and β chain which are both generated through random recombination of the α and β genes. β gene recombination involves 3 gene segments: variable (V), diversity (D), and joining (J) while α gene recombination involves V and J segments only. TCR β gene recombination occurs in a stepwise fashion in which D-to-J rearrangement is followed by V-to-DJ rearrangement (**Figure 2**) (52). Monoallelic rearrangement of the antigen receptors, termed allelic exclusion, involves a mechanism called feedback inhibition whereby further rearrangements of the TCRβ chain are prevented if a functional antigen receptor chain is produced. In the TCRα chain, rearrangement only ceases when the T cell undergoes positive selection. Therefore, multiple functional α chains can be produced and expressed on a cell surface but only one β chain. After successful VDJ recombination, T cells undergo positive and negative selection in the thymus to produce mature T cells that can recognize foreign antigen presented by HLA while sparing self-antigens (53). Thymic output stops in early adulthood after giving rise to around 10^6^ to 10^8^ unique T cells as identified by their TCR sequence. Such repertoire diversity underlies the breadth of the adaptive immune response against an array of immune targets. BCR diversity is generated *via* a similar V(D)J gene rearrangement process (54).

Considering this profound diversity and specificity, the identification of unique TCR and BCR sequences using lymphocyte receptor sequencing provides an important opportunity to characterize and monitor the dynamic changes in the immune repertoire in response to an antigenic stimulus, particularly HLA molecules, in organ transplantation. This review describes a systematic synthesis of current knowledge and methods for using lymphocyte receptor repertoire sequencing in the setting of solid organ transplantation. First, we summarize different sequencing approaches and compare their relative advantages in a clinical setting. Next, we review current data pertaining to alloreactivity and tolerance in renal transplant and the more limited data in other solid organ transplants. Finally, we discuss promising areas for future study in this space.

## 5 Methods

Two authors (P.W. and D.P.C) performed the searches in MEDLINE and PubMed Central, SCOPUS and EMBASE library databases. Commencing with T-cell and B-cell receptor sequencing, five refined key search terms were used in combinations for a total of six queries using the Boolean operator “AND”, as this allowed for only studies mentioning key search terms to be included (**Table 1**). Selected search phrases were submitted to PubMed on Oct 27, 2021. All searches were conducted with filters selecting articles that studied humans and English language articles published from January 1, 2010 – Oct 27, 2021. This date range was chosen due to the wide availability of high throughput sequencing post-2010. Total references returned includes replicates. Additional sources were identified by reviewing the reference lists of articles which met inclusion criteria. All studies identified were assessed to determine eligibility based on title and abstract. Potentially eligible studies were retrieved, and the full study report evaluated. Disagreements were resolved by consensus or adjudicated by a third reviewer (K.R.S).

**Table 1:** Selected search phrases submitted to PubMed on Oct 27, 2021.

Search Phrase	References returned
(Donor reactive) AND (T cell repertoire)	110
(Donor reactive) AND (B cell repertoire)	39
(T cell repertoire) AND transplant*	728
(B cell repertoire) AND transplant*	211
(T cell repertoire) AND clonal*	864
(B cell repertoire) AND clonal*	479
**TOTAL**	2431

All searches were conducted with filters applied for English language articles published from 2010-2021. Total references returned includes replicates. Truncation symbol used to retrieve search results for all words containing the phrase preceding *.

**Figure 3:**
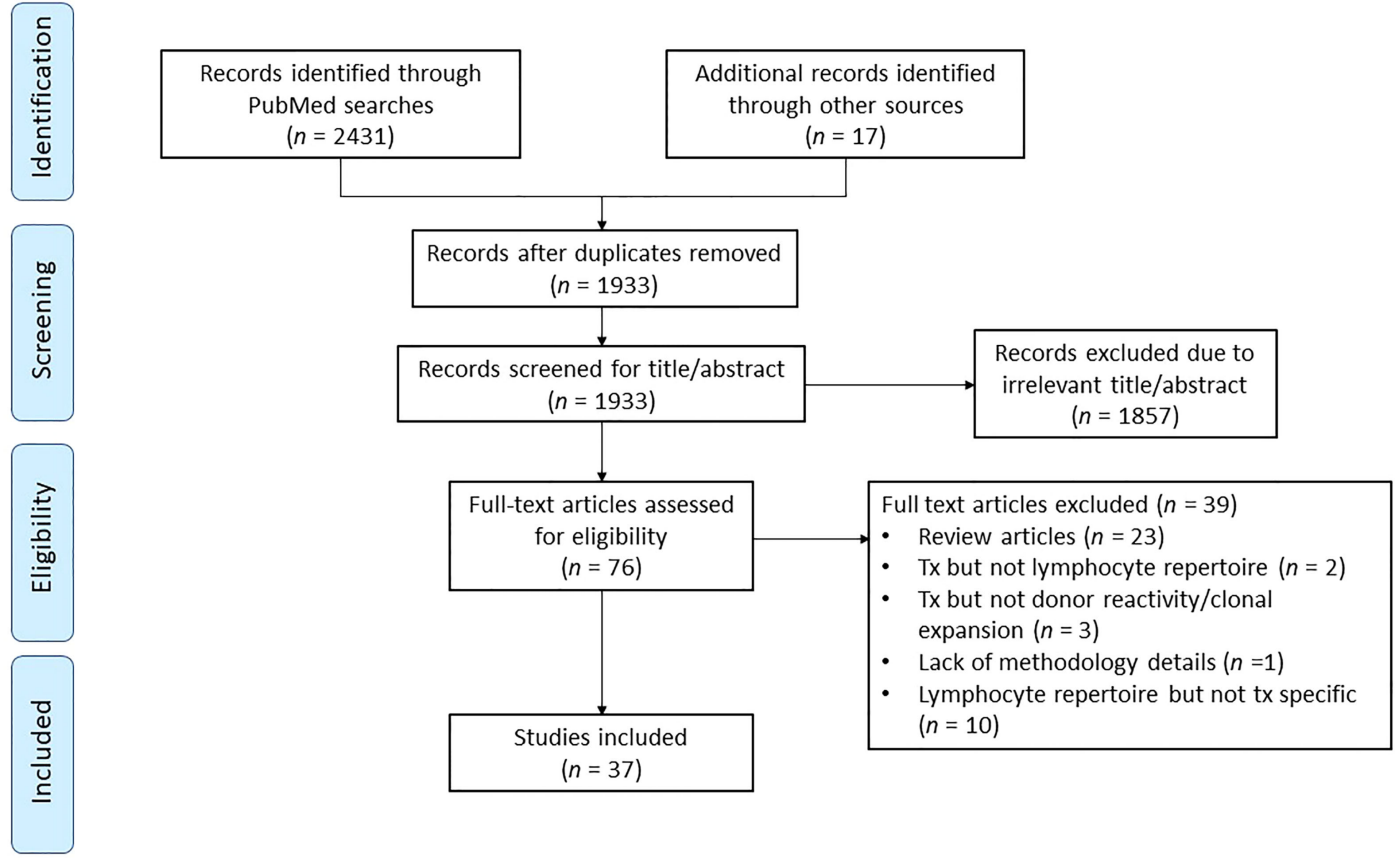
Flow diagram for records identified, included, excluded, and reasons for exclusion.

Articles were then evaluated for inclusion in the study according to the workflow process summarized in **Figure 3**. Once the studies were extracted and reviewed, those that applied TCR or BCR sequencing to solid organ transplant were selected for in-depth review. Studies that focused on non-human species were excluded due to disparities in the nucleotide sequences of receptor genes (55). Articles that focused on hematopoietic stem cell transplantation, cancer or other diseases which mentioned transplant but did not address immune monitoring, lymphocyte receptor repertoire or clonal reactivity were excluded. Likewise, full articles that lacked a detailed methodologic description were excluded from the review.

Study characteristics were categorized and summarized. Details included publication reference (first author, journal, year of publication, article type), receptor type (eg. TCR or BCR), organ transplant, sequencing region (eg. TCRβ sequencing), PCR method (eg. multiplex), starting material (eg. RNA), and whether a mixed lymphocyte reaction was conducted to identify clonal targets. For applicable studies, the subtype of immune cells investigated was noted. Other notable study characteristics were documented at the discretion of the reviewer.

## 6 Results

### 6.1 Targets and strategies for lymphocyte receptor sequencing

A summary of the studies included in this review and their characteristics are outlined in **Table 2**. TCR sequencing was performed more frequently than BCR sequencing in most studies (65% *vs* 35%), which could be due to the role of T cells in multiple mechanisms of graft injury, but perhaps also because there are established B cell methods including DSA testing. Not only are T cells involved directly in CMR, but they also play a role in the evolution of antibody-producing B cells that underly AMR. This points to the potential clinical utility of TCR sequencing in monitoring early phases of the alloimmune response with the goal of early intervention to prevent irreversible graft. BCR sequencing may also have clinical potential as it could provide further insight into the dynamics of AMR which is generally a more destructive form of graft injury.

**Table 2:** Summary of findings, methodology and demographic characteristics of included studies.

Author and Year	Cohort	Max Follow-up (months)	Genomic Target	PCR Method	Starting Material	Results	Limitations
Alachkar 2016 (2)	50 ktx	3	TCRβ CDR3	5’ RACE	RNA	Expansion of TCR repertoire at time of TCMR compared to pre-tx and 1m	Short follow-up, limited TCMR samples
Arrieta-Bolaños 2018 (56)	3 healthy	–	TCRβ CDR3	Multiplex	DNA	ND in clonality of alloreactive CD4+ against DPB1*02:01 or DPB1*09:01, alloreactive clones low in abundance	Public TCR clones may be alloreactive to DPB1 mismatch
Aschauer 2021 (57)	117 non-sensitized ktx, anti-CD25 induction	12	TCRβ CDR3	5’ RACE	RNA	DRC CD4 ↑ 0.72% to 1.89%, graft-infiltrating 6X circulating DRC	Short follow-up
Beausang 2011 (58)	19 highly-sensitized, 7 low-moderately-sensitized ktx	6	BCR IgHV	5’ RACE	RNA	Upon depleting B-cell therapy, ↑ proportion IgG/IgA. ↑ frequency mutation IgM/IgD post-tx	Small sample size, bulk RNA seq = cell-to-cell variability in expression level
Bellan 2011 (59)	6 ktx acute TCMR, including 3 AMR	20	BCR IgHV	5’ RACE	DNA	CD20+ B cells expressed highly mutated V genes without clonal expansion in TCMR alone or TCMR with AMR	Small sample size
Cappuccilli 2017 (60)	8 HD-Tx 7 HD 6 healthy	–	TCRβ CDR3	Multiplex	RNA	HD-Tx > HD > healthy in altered Vβ spectra	Spectra-alteration pattern is variable between individuals
Emerson 2014 (61)	6 healthy	3	TCRβ CDR3	Multiplex	DNA	Thousands of clones proliferate in MLC, alloreactive repertoire dominated by high abundance clones	Graft-infiltrating clones may not be detected by MLC
Ferdman 2014 (62)	21 ktx with graft failure	–	BCR IgHV	Multiplex	RNA	Clonal expansion and somatic hypermutation ↑ in graft than blood	Only 1 patient analyzed for comparative graft *vs*. blood
Gao 2017 (63)	4 ktx, 3 tolerant upon CKBMT	12	BCR IgHV	Multiplex	RNA	High frequency of transitional B cells and diversified repertoire during B cell recovery, memory B cell prevalent at 6m post-tx	Small sample size
Habal 2021 (64)	4 cardiac allograft vasculopathy at re-tx	–	TCRβ CDR3, BCR IgHV	5’ RACE	RNA	Extensive overlap in intragraft and blood TCRβ but minimal overlap in IgHV repertoire	Small sample size, *in vitro* PMA/ionomycin stimulation may modify T cell phenotype
Han 2020 (65)	34 liver tx (11 acute rejection, 23 stable), 20 healthy	3	TCRβ CDR3	Multiplex	RNA	↓ repertoire diversity in rejection cohort and distinct V gene usage	TCRβ repertoire characterization alone may not be enough to distinguish rejecting graft from stable
Huang 2019 (66)	39 IgAN, 13 non-IgAN, 60 healthy	–	TCRβ CDR3, BCR IgHV	Multiplex	RNA	IgAN had ↓ CDR3 length, ↑ IgA1 and hypermutation rate; TCRβ and IgHV clones can distinguish type of nephropathy	No TCRβ data on non-IgAN cohort, may require further studies on involvement of T cells in IgAN pathogenesis
Jones 2021 (67)	180 liver tx	12	TCRβ CDR3	Multiplex	DNA	↑ baseline repertoire clonality associated with sepsis 3m and 12m post-tx, but does not predict mortality	Baseline clonality determined pre-tx, thus repertoire changes post-tx may not be reflected
Kim 2021 (68)	1 hand tx	14	TCRβ CDR3	5’ RACE	RNA	↑ alloreactive GILs compared to blood; differential Vβ gene usage between graft and blood T cells	Small sample size, ex vivo expansion of GILs may alter clonal frequency
Lai 2016 (3)	10 ktx, 10 healthy	7 days post-tx	TCRβ CDR3	Multiplex	DNA	↓ diversity, ↑ high abundance clones in pre- and post-ktx compared to healthy, increased specific Vβ usage post-tx	Short follow-up
Lai 2019 (69)	3 ktx with acute rejection	7 days post-tx	BCR IgHV	Multiplex	DNA	↓ diversity, differential IgHV and IgHJ expression post-tx	Short follow-up, limited sample size
Leventhal 2015 (70)	19 CKBMT (12 tolerant and off IS)	18	TCRβ CDR3	Multiplex	DNA	Post-tx diversity of chimeric subjects similar to pre-tx; 97% unique clones post-tx, not present in pre-tx or donor; persistent chimeric patients developed infections while off IS	Only 9 samples had processed TCR data; no timepoints between tx and 2 years post-tx
Link 2016 (71)	4 HSCT	–	TCRαβ CDR3	–	RNA	Half of CD8+ repertoire dominated by CMV-specific T cells; ↓ diversity following CMV infection	Small sample size, CMV-specific CD8+ T cells defined by binding to restricted number of epitopes
Luque 2018 (72)	10 highly-sensitized, 5 non-sensitized	–	BCR Fluorospot	–	PBMC or B cells	Fluorospot assay capable of quantifying anti-HLA donor-reactive memory B cells	Further experiments comparing highly- and non-sensitized in ability to detect various anti-HLAs required
Mathew 2018 (73)	3 healthy	21 days	TCRβγ CDR3	–	DNA	CD40L-activated B cells stimulated AgTregs had ↓ diversity, ↑ polyclonality, demethylated Treg-specific demethylated region CNS2 at day 21 culture	Small sample size; polyclonal stimulation with CD3/CD28 may affect repertoire clonality
Moore 2020 (74)	4 cardiac allograft vasculopathy grafts at tx	–	BCR IgHV	Multiplex	DNA	B cell expansion, ↑ mutated rearrangements in graft but not blood	Small sample size
Morris 2015 (75)	4 CKBMT, 2 ktx	24	TCRβ CDR3	Multiplex	DNA	↓ post-tx donor-reactive clones in 3 CKBMT, ↑ donor-reactive clones in 2 ktx; ↓ diversity in non-tolerant patients	Small sample size, differences in IS between two cohorts
Nguyen 2017 (76)	3 EBV-positive lung tx	13	TCRαβ CDR3	Multiplex	DNA	TCRαβ repertoire biased towards TRVA-5; EBV-specific repertoire stable post-tx in absence or presence of EBV reactivation	Small sample size; repertoire changes not quantifiable due to limitations of bulk RNA TCR seq
Pineda 2019 (77)	27 ktx: 10 NP, 10 progressors (PNR), progressors with rejection (PR)	24	BCR IgHV	Multiplex	DNA/RNA	NP ↑ diversity, PR ↓ diversity; ↑ clonal expansion, presence of specific B cell clones and IGHV genes associated with ↑ risk of rejection	Sample highly selected set of pediatric patients
Pollastro 2021 (78)	1 healthy	10 days	TCRβ CDR3	Multiplex	RNA	*In vitro* expansion of moderate to frequently occurring clones 5-10 days post-stimulation; 15% clones showed ↑ frequency over 10 day culture; IL-4 addition induces expansion of IL-5 producing TCR clones	Short follow-up and sample size
Savage 2018 (79)	3 tolerant, 1 non-tolerant CKBMT; 3 healthy	6	TCRβ CDR3	Multiplex	DNA	ds-Tregs expanded 6m post-tx in tolerant patients, reduced in non-tolerant	Small sample size, large number of pre-tx cells required for assays
Savage 2020 (80)	3 tolerant, 5 non-tolerant liver tx	48	TCRβ CDR3	Multiplex	DNA	↓ donor-reactive TCRβ sequences in both tolerant and non-tolerant, magnitude of ↓ greater than non donor-reactive sequences	Lack of tissue or graft-infiltrating sample to confirm clonal deletion mechanism
Schober 2020 (81)	6 healthy CMV+	170 days	TCRαβ CDR3	Multiplex	RNA	Latent CMV infection resulted in ↑ proportion of larger T cells with low TCR affinity; clones with high affinity dominated acute immune response, low affinity preferred for chronic response	Small sample size; cannot rule out stochasticity as a factor of early/late repertoire dominance due to variability of single-cell-derived T cell populations
Smith 2016 (82)	10 SOT (6 lung, 3 kidney, 1 heart) following CMV-specific ACT	42 weeks	TCRβ CDR3	Multiplex	DNA	ACT responders showed ↑ CMV-specific clonotypes, ↓ diversity, ↑ effector memory T cells; no difference in TRBV usage was seen between responders and non-responders	Small sample size of non-responders (2/10)
Stranavova 2019 (83)	11 CMV+ HLA class I mismatched ktx	12	TCRβ CDR3	Multiplex	DNA	Association between pre-tx presence of CMV IE-1-specific T cells and acute rejection; shared TCRβ sequences between CMV IE-1 and donor-reactive T cells in pre-tx blood and post-tx graft	Lacking clonal frequency data of shared sequences and association with rejecting grafts
Vollmers 2015 (84)	12 heart tx (6 with rejection, 6 without)	15	BCR IgHV	Multiplex	DNA	Proof-of-concept IS monitoring: BCR seq diagnoses acute rejection *via* increased immune activity (71.4% sensitivity, 82% specificity with dd-cfDNA as standard)	Cohort is small and highly selected for rejection status and availability of longitudinal samples
Wang 2019 (85)	10 ESRD, 6 healthy	–	BCR IgHV	Multiplex	DNA	ESRD had ↑ clonal expansion, 5 upregulated and 9 downregulated VDJ genes; no difference in repertoire diversity was observed, although the distribution of ESRD was more skewed	Small sample size, differences in repertoire diversity not significant
Weinberger 2015 (86)	10 nephrectomy patients	–	TCR, BCR IgHV	Multiplex	DNA	Of graft-infiltrating clonotypes, 68% highly expanded T cells and 30% of the highly expanded B cells can be found amongst top 5 abundant clonotypes in blood; ↑ mean diversity in BCR repertoire compared to TCR across blood and kidney	TRBJ frequencies could not be compared to due primer limitations
Yan 2018 (87)	6 hepatitis B-related ACLF, 6 healthy	–	BCR IgHV	Multiplex	DNA	ACLF had ↑ clonal expansion, 6 upregulated and 19 downregulated VDJ genes; no differences in repertoire diversity	Small sample size
Yang 2018 (88)	6 liver tx, 6 healthy	7 days	TCRβ CDR3	Multiplex	DNA	↓ diversity in tx samples, with lowest at 7d post-tx; pre-tx had ↑ highly expanded clones compared to post-tx and healthy; ↑ TRBV expression in public TCRs post-tx	Short follow-up, given repertoire diversity was lowest at last timepoint, further timepoints could be insightful
Zhang 2021 (89)	30 CMV+, 25 CMV-	16	TCRαβ CDR3	Target enrichment	RNA	CMV+ associated with ↓ in naïve CD4+ T cells and ↑ CD4+ and CD45RA+ effector memory T cells, ↑ clonal expansion, and ↓ repertoire diversity	Only peripheral blood samples analyzed
Zuber 2016 (90)	11 intestinal tx, 7 rejectors	26	TCRαβ CDR3	Multiplex	DNA	Non-rejectors had donor T cells enriched for persistent GvH-reactive clones, while rejection was associated with ↑ HvG clones and ↑ T cell turnover	Role of *de novo* HvG clones in graft infiltration not assessed

ACLF, acute-on-chronic liver failure; ACT, adoptive T cell therapy; AMR, antibody-mediated rejection; CKBMT, combined kidney and bone marrow transplant; DRC, donor-reactive cells; ESRD, end-stage renal disease; GIL, graft-infiltrating lymphocytes; HD, hemodialysis; HSCT, hematopoietic stem cell transplantation; IgAN, IgA nephropathy; ND, no difference; NP, non-progressor; PNR, progressors without rejection; PR, progressors with rejection; TCMR, T cell mediated rejection. ↓, decreased; ↑, increased.

For purposes of identifying alloreactive clones, generation of donor-specific T or B cells could be a valuable resource. Mixed lymphocyte culture (MLC) coupled with receptor sequencing was a common method used amongst studies. Pre-transplant culturing of irradiated donor PBMCs with proliferation dye carboxyfluorescein diacetate succinimidyl ester (CFSE) stained recipient PBMCs over 5-7 days (79, 83, 91) generated an alloreactive fingerprint which could be longitudinally tracked post-transplant for immune monitoring. Morris et al. cultured donor PBMCs with purified T cells as responder cells (75). In terms of culturing conditions, cell plating concentrations were between 1-3 x 10^6^ cells/mL, with a 5-fold increase in frequency from seeding to harvesting defined as donor-reactive clones. Harvested cells were then fluorescence activated cell sorted (FACS) to isolate CFSE^low^ proliferating cells as well as CD4+, CD8+, and Tregs. Taken together, MLC approaches have been similar although with slight variations in culturing protocols.

The TCRβ chain was the genomic region sequenced in all 24 TCR sequencing studies, with 4 studies targeting both TCRα and TCRβ chains. Since each T cell can potentially express more than one α chain, but only one β chain, the β chain is considered a precise candidate to uniquely identify a given T cell clone (92). All studies sequenced the complementary determining region 3 (CDR3), a highly polymorphic region in TCRs and immunoglobulins.

Differing approaches were used to generate sequencing reads. Of the 25 studies that detailed PCR methods, 20 (80%) used a multiplex PCR method. This versatile approach accepts either gDNA or RNA input, but the mix of primers targeting only known V gene alleles means that novel alleles cannot be detected. It is also prone to amplification biases which can be mitigated with molecular barcoding (93). Five studies (20%) used nested PCR or 5’ rapid amplification of cDNA ends (RACE), the latter of which uses only RNA as starting material. 5’RACE preserves the 5’ end of the mRNA by incorporating extra nucleotides at the 3’ end of the cDNA molecule and a template-switch oligonucleotide. This enables the reverse transcriptase enzyme to replicate both templates completely and facilitate greater coverage of TCR variants. Ruggiero et al. developed a unique affinity target enrichment approach (94). RNA starting material was used to synthesize cDNA using a biotinylated primer targeting the constant (C) gene, which is adjacent to the rearranged VDJ genes. The cDNA fragments were magnetically captured on streptavidin beads for PCR amplification, allowing for small quantities of cDNA (up to 10 ng) to represent greater repertoire diversity.

### 6.2 Lymphocyte receptor sequencing in renal transplantation

Lymphocyte receptor sequencing has been applied most extensively in the context of renal transplantation to identify and track the specific alloreactive T cell repertoire, as well as characterize general trends in the peripheral and graft infiltrating T cell repertoire.

To define an ‘alloreactive T cell fingerprint’ Morris et al. developed a novel method using a MLC of recipient PBMCs labelled with CFSE and irradiated donor PBMCs labeled with Cell Tracer Violet for 6 days. The cultured cells were then FACS sorted for CD4+ and CD8+ CFSE-low cells and then their TCR β-chain CDR3 regions were sequenced. Putative donor-reactive clones are then defined as those that expanded at least 5-fold relative to their abundance in the baseline pre-MLC recipient repertoire (75).

This approach was then used to monitor T cell alloreactivity and identify mechanisms of tolerance in 4 CKBMT patients in whom immunosuppression (IS) was withdrawn, and 2 conventional living donor renal transplant patients. In the two conventional renal transplant patients CD4+ but not CD8+ donor reactive T cells increased in the post-transplant peripheral repertoire over time. In these patients, the magnitude of the *in vitro* alloreactive response, in terms of diversity and expansion of clones, correlated well with the magnitude of the alloreactive response post-transplant. CKBMT patients who developed tolerance had a significant reduction in both number and diversity of circulating donor reactive T cells while those who experience rejection after IS withdrawal did not. This supports a role for clonal deletion as a mechanism of tolerance (75).

Savage et al. then leveraged TCR sequencing to study the role of T regulatory cells in maintaining tolerance in the same CKBMT patient population which included 3 tolerant and 1 non-tolerant subject after 10 months of IS withdrawal. They used FACS sorting for CD4+CD25+CD127- Treg lineage markers and TCR sequencing to define Treg repertoires under 3 different conditions: unstimulated, after stimulation with activated donor B-cells, or after stimulation with MLC in the presence of CTLA4Ig. The ‘activated donor B-cell method’ identified the most biologically relevant repertoire of Tregs that correlated best with tolerance by clonal fraction and cumulative frequency at 6 months. Tregs identified by this method were not expanded in the non-tolerant subject suggesting that tolerance despite lack of persistent mixed chimerism in these CKBMT patients may be in part explained by T regulatory cells (79).

These studies not only highlighted how TCR sequencing can be used to elucidate the biology of tolerance in specialized renal transplant cases, but also provide a basis for further studies using TCR sequencing of the alloreactive repertoire as a biomarker in conventional renal transplant. Aschauer et al. leverage a similar method in a single-center prospective cohort study designed to evaluate the expansion or deletion of alloreactive T cells in the post-transplant repertoire of patients with or without acute TCMR. Secondary outcomes included local T cell expansion in the graft during a rejection episode and changes in clonal diversity over time in rejecting versus non-rejecting patients (95). Using a cohort of 12 patients (3 with TCMR, 3 with borderline rejection, and 6 control patients), they showed that circulating CD4+ alloreactive T cells increased after transplant with no significant difference between controls and rejectors, even at the time of rejection. The biopsy infiltrating repertoire, however, was enriched for donor-reactive T cells at the time of rejection relative to controls (91).

In a separate study aimed at understanding the impact of different induction therapies on the alloreactive repertoire in 5 patients with pre-formed DSA induced with rabbit anti-thymocyte globulin (rATG) plus immunoadsorption and 5 without DSA given Basiliximab. MLC and TCR sequencing was performed at baseline and the alloreactive repertoire tracked for a year. They showed that over 1 year the percentage of peripheral CD4+ but not CD8+ donor-reactive T cells increased at similar frequencies across both groups (57). While these data do not shed further light on the diagnostic utility of TCR sequencing it suggests that there is no significant difference in T cell alloreactivity associated with either induction method alone.

The remaining studies in this space aim to measure general features of the T and B cell repertoire in patients prior to and after transplant. The conclusions that can be drawn from these studies as to the clinical utility of TCR sequencing in transplant are limited by a lack of systematic approaches and standardized metrics.

Two studies examined trends in the T cell repertoire of patients both pre- and post- renal transplant who do experience rejection. Lai et al. characterized the peripheral T cell repertoire in 10 transplant recipients preoperatively, then on postoperative day (POD) 1 and POD 7. These were then compared to a cohort of 10 healthy controls at a single time point. Repertoire overlap between patients, also known as the public repertoire, was minimal. High-abundance clones in individual patients were present at all time points suggesting that abundant clones can be reliably tracked in the peripheral circulation of an individual over time (3). No difference was observed in CDR3 length, VD indel length and DJ indel length between controls and transplant recipients at any time point. Repertoire diversity is a marker of a versatile immune system able to respond to multiple stimuli and is known to be restricted in the setting of autoimmune pathology such as lupus, Crohn’s, and psoriasis (96). In this study, diversity was highest in controls and lower in transplants patients at baseline, decreasing further by POD 1, then again by POD 7. While it is difficult to infer the biological significance of this, one might hypothesize that this decreased diversity reflects the relative immune dysfunction previously described in patients with ESRD (97) that is further aggravated by either lymphodepletion or immunosuppression in transplant. Alachkar et al. used a prospective multi-center trial design to study peripheral and graft infiltrating T cell repertoire metrics in renal transplant patients with and without rejections in the first 3 months after transplant. TCR diversity was lower in graft samples than peripheral samples, as expected, but otherwise there was no unifying pattern of TCR diversity in individual patients over time, and no difference in diversity between rejectors and non-rejectors (2). However, this study went on to define recurrent highly abundant clones as the top 10 most frequent clones in each repertoire that occur at all timepoints in both blood and biopsy specimens. They show that these clones were expanded in the peripheral repertoire of transplant patients at the time of rejection but not time-point-matched non-rejectors. They were also able to show in one patient, that 6 of the top 10 most abundant clones infiltrating the graft at the time of TCMR could be identified in the peripheral repertoire of the same patient at multiple earlier time points. The hypothesis here is that highly abundant clones present in both the graft and the peripheral repertoire after transplant are more likely to be alloreactive and therefore interesting from a diagnostic perspective although this cannot be confirmed from these data.

The presumption that graft-infiltrating clones occur in peripheral blood underlies the use of TCR sequencing as a monitoring tool in renal allograft rejection. Weinberger et al. attempted to quantify this using nephrectomy samples from 10 patients of whom one was a transplant patient suffering from acute T cell mediated rejection. They showed poor clonal correlation between the B and T cell receptor profiles of tissue and peripheral samples, but that the correlation was greatest among the top 20 most abundant T cell clones (86). There was however no correlation in frequency between graft-infiltrating and peripheral T cell clones even among the top 20 most abundant clones.

While these studies that quantify general TCR repertoire metrics in renal transplantation suffer from heterogenous study designs, low patient numbers, and highly variable approaches to data analysis, they offer initial guidance to the feasibility and potential of this technology in a clinical setting and several key technical insights. First, TCR repertoire diversity, CDR3 length, and gene usage are all commonly used metrics in this setting. Second, highly abundant clones in the repertoire can be more reliably tracked over time for a given patient. Third, the graft infiltrating profile correlates best with the peripheral repertoire for highly abundant clones but that this correlation is not quantitative. Future studies will have to better define the nature of highly abundant clones and delineate their cognate targets to better understand their role as a clinical tool in renal transplant.

Similar work has also been conducted in B cell repertoire (BCR) sequencing. Pineda et al. performed longitudinal BCR sequencing in renal transplant patients, dividing them into three cohorts consisting of: patients who did not reject and did not sustain chronic graft damage, patients who did not reject but did sustain chronic graft damage, and patients who did reject and sustain chronic graft damage (77). This study showed that higher BCR diversity prior to transplant correlated with the development of rejection, while patients who ultimately rejected their graft had a greater decrease in BCR diversity and increased clonal expansion after transplant. While reduced diversity may be explained by intensification of immunosuppression in rejecting patients, the authors comment that this phenomenon was observed at time-points preceding rejection pointing to the possibility of a causal link. Perhaps the most interesting finding of this study for future investigation is the association between specific IGHV gene use and rejection. For example, the *IGHV3-23* gene was used significantly more in patients who develop rejection across all time-points (77), in line with prior studies implicating this gene with transplant rejection (98). Moreover, the clonal antibodies arising from this specific IGHV gene, and others found to be clonally expanded in transplant, are known to react with bacterial pathogens and not allogeneic HLA (99). While the cause of this focused clonal expansion remains to be determined, it points to the preferential selection of specific clones over time by an unrelated powerful antigenic stimulus that could in turn contribute to transplant immune mediated pathology. It also raises the potential that certain common antigens are responsible for driving rejection. Further work in this area promises to yield not only potential biomarkers to risk-stratify patients for rejection, but also new insights into the biology of rejection.

### 6.3 Lymphocyte receptor sequencing in other solid organ transplants

TCR sequencing has also been evaluated in other solid organ transplant studies including liver, intestinal, and heart transplant.

Two studies examined the role of TCR sequencing in liver transplant. Yang et al. investigated general differences in TCR repertoire dynamics between liver recipients and healthy controls (88). TCR sequencing of 6 liver transplant patients pre-transplant and post-transplant day 1 and 7 revealed shared TCR clones between these time points. Few CDR3 sequences were shared amongst individuals, but more were shared amongst healthy people than amongst patients. The distribution of CDR3 and VD/DJ indel length was found to be similar in all controls and patient groups. Clones with a greater than 0.5% frequency were defined as highly expanded clones, but only 10-15% of the total TCR repertoire was found to consist of highly expanded clones, translating to an average of 7.2, 6.8, and 2.3 clones in the pre-transplant, POD1, and POD7 groups respectively. This highly expanded population showed little similarity in DNA or amino acid sequence but had shared V-J gene combinations. The degree of expansion of most expanded clones was highest on POD7 (p=0.046). Transplant patients were found to have lower diversity than controls, with significantly decreased diversity on POD7 compared to other groups (88). Savage et al. identified donor-reactive clones by first performing a pre-transplant MLC followed by post-transplant TCR sequencing to track donor-reactive clones in 8 liver allograft recipients (80). Amongst three tolerant and five non-tolerant recipients, all showed significant reductions in donor-reactive TCRβ sequences post-transplant. This is in contrast to data from conventional kidney transplant recipients that shows an increase in alloreactive clones post-transplant (100). Compared to donor-reactive clones, non-specific TCRβ sequences identified in the recipient repertoire by random third-party MLC did not show a distinct pattern of clonal expansion or contraction. Taken together, these data show that TCR sequencing can not only be used to identify general alterations in the T cell repertoire after liver transplant, but also that it can be applied to specifically track donor reactive clones in these patients.

Intestinal allografts are rich in lymphoid tissue, which over time engage in a bidirectional exchange with the recipient in which various degrees of bone marrow and peripheral blood chimerism occur or the allograft can become repopulated with recipient lymphocytes. Two detailed studies from the Sykes group leverage TCR sequencing to understand the biology of this unique case study in SOT. First, using MLC and TCR sequencing they define a graft-versus-host (GvH) and a host-versus-graft (HvG) alloreactive T cell profile (90). Instances of intestinal allograft rejection were correlated with intra-graft donor repertoires enriched for HvG-reactive clones and accelerated T cell turnover, whereas low levels of intra-graft recipient chimerism were associated with immunologic quiescence (90). Furthermore, this study challenges the paradigm that during rejection most graft infiltrating lymphocytes are not allograft specific by showing that up to 80% of CD4 and CD8 T cell clones present in allografts experiencing early cell mediated rejection. They then expand on this study to show that CD8 cytotoxic GvH clones migrating from the graft-lymphoid tissue home to the bone marrow where they create space in the stem cell niche for donor derived hematopoetic stem cells which in turn facilitate bone marrow and peripheral chimerism and graft tolerance (101).

BCR sequencing has been used to monitor the response to immunosuppression in a cohort of 12 heart transplant recipients of whom 6 participants had moderate-to-severe acute rejection (84). This study first set out to characterize immunoglobulin heavy chain (IGH) dynamics in the absence of rejection. IGH transcripts were measured immediately post-transplant then at 6 months after prolonged immunosuppression and categorized into low- and high- expression transcripts. Low-expression transcripts were represented by only one type of Ig molecule and likely expressed by naïve or inactive B-cells. High-expression transcripts were represented by more than one molecule and likely expressed by an activated B cell. While the total number of high-expression IGH transcripts detected did not change between timepoints, the contribution of high-expression IgA, IgE, IgG, mutated IgM sequences diminished relative to total transcripts at late time points and inversely correlated with trough levels of Tacrolimus and Annelovirus load, both of which are previously described markers of immunosuppression (102). Based on these findings the authors defined an activated B cell sequence (ABS) metric as the ratio of highly expressed IgA, IgD, IgE, IgG, and mutated IgM sequences to total sequences. ABS not only inversely tracked with degree of immunosuppression, but the median ABS level rose by 3-fold in the setting of rejection as diagnosed by endocardial biopsy and dd-cfDNA. Moreover, a gradual and significant increase in ABS level was observed in the 4 months leading up to the biopsy proven rejection event, highlighting the potential of this method for early detection of rejection. Last, this study looked at two specific case studies and shows that ABS was also increased in a heart transplant patient with recurrent opportunistic infections, and another who received Filgrastim in the setting of a subsequent bone-marrow transplant for AL amyloidosis (84).

### 6.4 Infectious complications of solid organ transplant

Opportunistic infections are not only a serious complication of immunosuppression in solid organ transplant but also predispose patients to immune alloreactivity. Common infectious pathogens include Cytomegalovirus (CMV), Epstein-Barr virus (EBV), BK polyoma virus, Varicella zoster virus (VZV), Herpes simplex virus (HSV), and Pneumocystis jirovecii. Lymphocyte receptor sequencing has the potential of offering insight not only into the risk and dynamics of opportunistic infections in solid organ transplant, but also the mechanistic underpinnings of how viral infections predispose patients to immune rejection.

CMV is a common viral infection that is associated with allograft rejection, although the mechanistic underpinning of this phenomenon is poorly understood. TCR sequencing offers a unique lens through which to interrogate the hypothesis that cross-reactivity of T cells directed at alloantigen and CMV underlies this phenomenon. Using an observational cohort study design, Stranavova et al. showed a significant association between CMV immediate-early protein 1 (IE-1) specific T cell activity by IFNγ ELISPOT and acute renal allograft rejection and function (83). These data confirmed the well-established link between these two pathologies. They followed this with a study of 11 CMV-seropositive and HLA class I mismatched but low immunologic risk renal transplant patients. Using recipient PBMC cultures stimulated with donor cells or CMV antigens they identified several TCR beta sequences that cross-reacted to donor antigens and CMV. Biopsy specimens were available for TCR sequencing in 7 patients. CMV or donor-reactive clones identified from the previous mixed lymphocyte/antigen cultures were found in 6 out of 7 biopsy specimens and 3 of 7 biopsy specimens contained cross-reactive clones. Importantly, the highest number of cross-reactive clones was observed in patients with CMV reactivation with concomitant cellular rejection (83). While these numbers are low, and the absolute number of cross reactive clonotypes are small, these findings support a contributory role for cross-reactive clones in the pathogenesis of CMV associated rejection.

EBV is a common virus that seldom causes pathology in healthy immunocompetent adults. CD8 T cells provide important protection against EBV. GLCTLVAML (GLC) is a highly conserved immunogenic EBV peptide targeted by a restricted T cell repertoire that is highly conserved between individuals. Nguyen et al. leveraged TCR sequencing to quantify clonotypic diversity and abundance of T cells targeting GLC in healthy individuals, then tracked the dynamics of CD8+ GLC-specific clones over time in a cohort of lung transplant recipients before and after EBV exposure. They showed that, on average, the GLC-specific CD8+ TCRαβ repertoire consisted of 10 TCRαβ clonotypes per individual, and that this repertoire was stable before and after immunosuppression in lung-transplant patients without EBV reactivation. In the setting of clinical EBV reactivation, GLC CD8+ T cells expanded in the transplant recipients. There was marked overlap between repertoire at different time points, although the dominant clonotype changed with the onset of profound viremia (76). Another immunodominant EBV epitope, BRLF1, was shown to interact more with TCRα CDR3 sequences than those of TCRβ (103). Further studies are required to understand the immune repertoire changes in SOT associated with EBV and EBV-specific T cell dynamics during rejection.

Reactivation of BK polyoma virus, and BK nephropathy, in the setting of immunosuppression is another important infectious cause of graft loss which is currently best diagnosed with histology. Histological features of BK nephropathy can however mimic those of acute cellular rejection. This is further complicated by the possible co-existence of these conditions. Treatment of acute rejection involves increasing immunosuppression transiently which would worsen BK viremia and BK nephropathy as T cell mediated cellular immunity is the key to controlling BK infection. Indeed, TCR sequencing and flow cytometry with cell-sorting was used to show that BK viral clearance in transplant patients correlates with the repertoire diversity of BK virus specific T cells and markers of exhaustion in BK specific CD4+ T cells (104). This method to evaluate BK viral infection was developed into a diagnostic tool by Dziubianau et al. who used mixed lymphocyte or antigen culture to define an alloreactive and BK reactive T cell repertoire in 5 transplant patients, then evaluated the abundance of these clones in biopsy specimens and urine. They were able to detect BK directed T cell clones in biopsy and urine specimens of two patients with clear biopsy proven BK nephropathy. In one patient with proven BK nephropathy who did not respond to IS withdrawal, alloreactive clones were detected at higher frequency in biopsy and urine specimens suggesting acute cellular rejection to explain this phenomenon. Last, they showed that in chronic cytotoxic nephropathy neither type of clone was detected in either biopsy or urine specimens (105). Excitingly, this approach was used prospectively to influence treatment decision in a patient with a rising creatinine levels 6 weeks after living related donor kidney transplant in the setting of BK viremia and a biopsy consistent with acute TCMR negative for SV40 antigen. Donor and BK virus specific CDR3 sequences were identified by mixed cultures, and then quantified in the T cell repertoire from a graft biopsy. BK virus specific clones dominated the graft repertoire occupying 81.6% of clones. Based on this finding the clinical team tailored their treatment accordingly leading to resolution of the BK viremia and lasting improvement of graft function at 2-year follow-up (104). This work highlights the role of TCR sequencing as an adjunct diagnostic modality in complex differential diagnoses and shows the feasibility of detecting and tracking BK-specific clones in patient urine.

VZV, HSV, and Pneumocystis jirovecii are less studied in SOT through immune receptor sequencing methods. VZV complications are uncommon as most recipients are seropositive (106), but herpes zoster complications are more frequent and may include neuralgias and disseminated zoster. HSV causes more severe clinical manifestations and a slower response to therapy in SOT patients compared to immunocompetent individuals (107). Pneumocystis jirovecii risk is greatest in the first 6 months post-transplant and is higher for those who are seropositive for CMV and/or have low lymphocyte counts (108). Infection has been correlated with higher risk of graft failure and mortality regardless of rejection (109). Prophylactic measures are important in mitigating complications from these infections, giving potential for exploratory TCR/BCR sequencing studies to monitor viral-specific clones.

## 7 Discussion

In this article, we present a first, formal review of lymphocyte receptor sequencing in SOT to monitor alloreactivity, to describe how the technique is currently being used to monitor renal, liver, intestinal, and heart transplants, and to explore its potential in furthering our understanding of opportunistic infections and immune rejection.

From a methodologic standpoint, sequencing of the CDR3 region of the TCRβ locus was the preferred method for quantifying the T cell repertoire. In both renal and liver transplant recipients, broad alterations are observed in the T cell repertoire with decreased repertoire diversity relative to controls, minimal public repertoire overlap between patients, and reproducible overlap in private repertoire over time (2, 3, 88, 95). Furthermore, MLC and TCR sequencing can be applied to identify putative alloreactive clones and track them over time to evaluate both deletional and Treg induced tolerance among CKBMT and liver transplant patients (75, 79, 80). These studies however lack generalizability as they evaluate very specialized cases in solid organ transplant. The one available prospective study applying this technology to a standard renal transplant cohort is an excellent proof of feasibility and supports a potential role in monitoring alloreactivity but is inconclusive due to low enrollment and short-term follow-up (91). Studies on the effects of lymphodepletion on the T cell repertoire have been in consensus on an increase in donor-reactive CD4+ frequency; one study that investigated repertoire changes in rATG and Basiliximab induced patients found similar increases in alloreactive CD4+ but not CD8+ T cell frequency, supporting an earlier study that found patients receiving ATG showed post-depletional CD4+ CD25+ Treg and CD4+ effector memory phenotype (57, 83). This emphasizes the importance of CD4+ T cells in the alloimmune response and the need to focus monitoring efforts on them. Beyond alloreactivity, several case reports detail the utility of TCR sequencing in studying infectious complications of SOT. Generally, these show that CMV-reactive clones are associated with rejection events (83), and that EBV and BK polyoma virus directed clones can be used to distinguish between alloreactivity and viral injury as well as track viremia (104, 105, 110).

BCR sequencing was less commonly studied and only applied to describe trends in the repertoire before and after transplant. These studies showed an association between specific IGHV gene sequences and class-switched or highly mutated IgM sequences and rejection (77, 84). Somatic hypermutation, the accumulation of point mutations in the Ig variable region, was found to be higher in the graft than in blood and correlated with B cell expansion (62). This presents a further complexity in understanding AMR through BCR sequencing, as detected BCR sequences may have undergone mutation by the time the B cell has infiltrated the graft tissue. Thus, longitudinal tracking of B cell clones may be hindered by somatic hypermutation. No studies were found to have applied BCR sequencing to specifically quantify alloreactivity. However, this could be due to the presence of DSA testing which is an established method already used to detect B cell alloreactivity. This may also explain why there are fewer BCR than TCR studies in transplant overall as DSA testing may be a more efficient method to attain a similar goal. However, BCR sequencing could still hold value in providing earlier information on antigen specificities, which will be discussed later.

Practicality and financial considerations cannot be understated when considering a clinical assay. A TCR/BCR sequencing kit from private biotech companies can cost upwards of several thousand dollars, and sequencing services can cost even more. Meanwhile, Enzyme Linked Immunosorbent Spot (ELISPOT) kits can be more cost-effective (Abcam). In terms of assay time required, ELISPOT can take up to several days for optimal cytokine secretion. Although the initial MLC to establish the alloreactive repertoire can take several days, subsequent receptor sequencing procedures for routine monitoring would only require 2 days. Using nanopore sequencing, this time can be further decreased. While the cost-effectiveness of TCR/BCR sequencing assays may be improved upon as the technology matures, it shows promise as an efficient yet precise monitoring technique for SOT.

An immediate question this research poses is whether tools are available to predict antigen specificity based on TCR or BCR sequences. Studies on antigen prediction are limited, but two studies provide a glimpse for its potential. Grouping of lymphocyte interactions by paratope hotspots (GLIPH) is an algorithm developed by Glanville et al. that defines TCR specificity groups based on a dataset of MHC tetramer-sorted cells with structural data (111). It is also able to define specificities shared between TCRs and individuals, allowing for analysis of T cell responses based on the number and size of specificity clusters. TCRMatch is a TCR specificity analysis tool based on known epitope specificities in the Immune Epitope Database (IEDB) (112). It takes TCRβ CDR3 sequences as input and gives a specificity score for those with a match in the database. These tools could add to future work in immune repertoire sequencing in transplant. However, current databases are developed using well-studied antigens such as common viral and bacterial motifs. Given the complexity conferred by HLA epitopes, predicting how individual repertoires recognize them will require further studies.

Despite the scope and depth of this review, several limitations remain regarding translation to clinical diagnostic practice. First, the conclusions we can draw are limited by the quality of the evidence. While we have performed a comprehensive review of the literature, the studies on this topic are small observational cohort studies and case reports. These studies by their very nature are hypothesis generating. Second, prior to the advent of TCR sequencing, ELISPOT has been used to quantitate the allo-specific memory T cell response. While these data are beyond the scope of this paper, ELISPOT has shown promise in some clinical trials (110, 113, 114), and is another tool with clinical potential for alloimmune risk-stratification and monitoring. Third, the information we can garner from bulk TCR sequencing is limited in two respects. Even in the setting of MLR followed by TCRβ CDR3 sequencing, the antigen specificity of the putative alloreactive clones cannot be determined. Furthermore, once a putative alloreactive clone has been identified, the T cell subtype and phenotype of the clone cannot be ascertained with this method. Single-cell-level cell-surface markers and gene expression data have the power to achieve this. While beyond the scope of this review, early data leveraging this technology in transplant has been reported (101, 115), and provides a promising new direction for the field. And fourth, perhaps one of the most critical points, is that differences in immune activity between the graft and in circulation cannot be overlooked. BCR repertoire overlap has been shown to be small in some instances (64) and number of alloreactive cells and Vβ gene usage can differ between the two sites (68). Thus, sampling site could be a major source of variability in an immune repertoire sequencing assay.

Taken together, the findings outlined above point to the promise of lymphocyte receptor sequencing in detecting and monitoring the early phases of allorecognition and rejection in a clinically relevant setting. While the data is promising, larger prospective studies in a more generalizable SOT population are needed.

## Author contributions

KRS and PAK conceived the study idea and PAK obtained research funding. PW developed a search protocol and conducted the review. PW and DPC extracted the data. KRS and PAK supervised the study progress and provided regular feedback. PW and DPC wrote the draft of the manuscript. RS-P and CP were contributing members of the TCR research planning team. All authors contributed to the article and approved the submitted version.
